# Direct-Ink-Written Interdigital Capacitive Sensor on Beech Wood for Surface Moisture Monitoring: Electrostatic FEM Design and Proof-of-Concept Characterization

**DOI:** 10.3390/s26216968

**Published:** 2026-11-04

**Authors:** Paul Reber, Philipp Kersten, Sören Majcherek, Sören Hirsch

**Affiliations:** Department of Engineering, Technische Hochschule Brandenburg, Magdeburger Str. 50, 14770 Brandenburg an der Havel, Germany; philipp.kersten@th-brandenburg.de (P.K.); soeren.majcherek@th-brandenburg.de (S.M.); soeren.hirsch@th-brandenburg.de (S.H.)

**Keywords:** printed electronics, direct ink writing, interdigital capacitor, wood moisture content, finite element method, structural health monitoring

## Abstract

Moisture limits timber durability, and wetting begins at the surface. An interdigital capacitor (IDC) was printed by direct ink writing (DIW) of a silver paste onto beech wood, the substrate and sensing dielectric. The electrodes were designed within the smallest printable line width (210 μm) with an electrostatic finite element method (FEM) model; 90% of the sensitivity lies within 0.2 mm of the surface. One 24-finger sensor (width and spacing 300 μm) was characterized at 1–100 kHz over a 15-day sorption cycle (30–90% relative humidity (RH), 23 °C). Reference capacitors revealed a meter gain error of 22% at 100 kHz; after calibration, an identically printed glass reference agreed with the two-dimensional model within 3% (same session). The calibrated beech capacitance at 100 kHz rose from 7.0 to 13.7 pF, by 0.11 pF per percentage point of RH (short-term, noise-limited 3σ resolution 0.6–3.5% RH). Referred to moisture content (MC) assigned from tabulated equilibrium values, this is 0.47 pF per percentage point, close to the predicted 0.48 pF; the absolute values lie 5–17% below the model. The step response is two-phase. No gravimetric calibration was performed; the RH response is the primary result of this single-sensor study.

## 1. Introduction

Timber is regaining importance as a structural material, and its long-term performance is governed by moisture [1]: changes in moisture content (MC) cause swelling and shrinkage, and sustained high MC enables decay. Moisture monitoring uses resistance [2] or capacitive probes [3], interdigital electrodes on a circuit board placed on the wood [4], multi-wavelength planar sensors probing different depths [5], and electrodes embedded in glued-laminated timber [6]. Electronics on the wood itself include laser-induced graphene [7], resistive sensors from laser-graphitized lignin ink [8], screen-printed and vapor-deposited circuits [9], interdigitated moisture sensors on spruce, inkjet-printed on a primer or screen-printed and read out by impedance [10], and pencil-drawn graphite interdigital capacitors (IDCs) on pine [11]. In printed humidity sensors, paper has served as the active dielectric of IDCs [12], whereas foil-based IDCs rely on the sorption of the foil [13]. Direct ink writing (DIW) of viscous pastes [14] is maskless and needs no screen or primer, which makes it a candidate for rough, porous wood.

This paper combines DIW of a silver IDC on solid beech with an electrode design by field simulation within the line-width limit of the process and compares the measured response with the model. It contributes (i) a numerically verified finite element method (FEM) unit-cell model with a design map and the depth distribution of the sensitivity; (ii) the fabrication of the layout within a process window from a $2^{6-2}$ screening; and (iii) a 15-day sorption campaign at four frequencies with an identically printed glass reference and a calibration of the measurement chain with reference capacitors, giving the static and dynamic response, hysteresis, and reversibility of one sensor (proof of concept).

## 2. Materials and Methods

### 2.1. Substrate, Ink, and Printing

Cylindrical beech samples (*Fagus sylvatica*, 40 mm in diameter, 15 mm thick) were cut so that the printing face is a radial surface and sanded with P240 grit; the fingers were printed parallel to the grain. The silver paste FS0142 (ACI Materials, Ventura, CA, USA) was deposited by DIW on a NOVA platform (Voltera, Kitchener, ON, Canada) through a 100 μm nozzle (Subrex, Carlsbad, CA, USA) at 40 μm standoff, 300 mm/min, and a dispensing pressure of 600 (device units) in a single pass, and cured at 150 °C for 10 min. These settings are the process window of a $2^{6-2}$ fractional factorial screening (resolution IV, 16 runs) over species, grain direction, roughness, speed, pressure, and curing temperature (100/150 °C) [15]; curing at 150 °C is mandatory for continuous lines. The sheet resistance is $R_{□}\approx 15.1\;m\Omega /\mathrm{sq}$, the confocally measured film thickness t=12.0±2.2 μm (24 profiles, one sample), and the effective resistivity $R_{□}\;\cdot \;t\approx 1.8\;\times \;10^{-5}\;\Omega cm$. The smallest mean line width was 211 μm with a standard deviation of 12.4 μm over 20 test lines printed with the same settings (coefficient of variation 5.9%; the fingers of the sensor were not measured individually), so $w_{\min}\approx 210\;\mu m$ was adopted as the limit for finger width and spacing. A systematic width deviation of this size at the fixed pitch of 600 μm would change the base capacitance and the absolute sensitivity by about ±3% (Equation (2)), but not the relative response; random line-to-line variation averages over the 23 gaps (about ±0.6%).

### 2.2. Electrostatic FEM Model

At readout frequencies of at most 100 kHz, the structure is electrically small, so the field is quasi-static: in the charge-free dielectric, $\nabla \;\cdot \;\vec{D}=0$ with $\vec{D}=\varepsilon_{0}{\underline{\underline{\varepsilon }}}_{r}\vec{E}$ and $\vec{E}=-\nabla \Phi$ gives

(1) $$\nabla \;\cdot \;\left(\varepsilon_{0}\;{\underline{\underline{\varepsilon }}}_{r}\;\nabla \Phi \right)=0.$$

Equation (1) is solved with the two-dimensional electrostatic solver of Ansys Maxwell (Electronics Desktop 2025 R2, Ansys, Canonsburg, PA, USA) with fixed electrode potentials as Dirichlet conditions (the capacitance does not depend on their value) and $E_{n}=0$ at the outer boundaries of the air and wood regions, each 5 mm high; the capacitance follows from the stored energy $W_{e}=\frac{1}{2}\int_{V}\vec{E}\;\cdot \;\vec{D}\;dV$ as $C=2W_{e}/U^{2}$. A periodic unit cell of width p=w+s between two finger centers is modeled (Figure 1b; $E_{n}=0$ at its lateral boundaries); a comb of *n* fingers of length *l* then has $C=\left(n-1\right)\;C_{\mathrm{cell}}^{\prime }\;l$. The electrodes are rectangles of the measured thickness t=12 μm on a homogeneous wood half-space with a scalar permittivity $\varepsilon_{r}$; since the fingers run parallel to the grain, the field lies in the radial–tangential plane, and the two-dimensional solution depends only on $\sqrt{\varepsilon_{R}\varepsilon_{T}}$: scaling the depth coordinate by $\sqrt{\varepsilon_{R}/\varepsilon_{T}}$ maps the anisotropic Laplace equation in the wood onto the isotropic one with $\varepsilon_{r}=\sqrt{\varepsilon_{R}\varepsilon_{T}}$ and leaves the electrode plane y=0 and the normal flux through it unchanged, so the result is exact for a homogeneous half-space; with an additional shear, it holds for any orientation of the R and T axes in the cross-section ($\varepsilon_{r}=\sqrt{\det{\underline{\underline{\varepsilon }}}_{r}}$), i.e., independently of the growth-ring angle. For this mean, the tabulated transverse value is used [16]. The mesh is refined adaptively at the electrode edges until the stored energy changes by less than 1% between passes. The depth distribution of the sensitivity (Section 2.3) was computed separately from a two-dimensional finite-volume solution of Equation (1) for the same unit cell with infinitely thin electrodes on a nonuniform grid; refining the grid spacing at the surface from 5 to 1.25 μm changed the depth fractions by at most 0.004 mm, and the resulting $C_{\mathrm{cell}}^{\prime }$ lies within 0.5% of Equation (2). As an independent reference, the closed-form solution for infinitely thin, periodic electrodes on a homogeneous half-space [17,18],

(2) $$C_{\mathrm{cell},\mathrm{an}}^{\prime }=\varepsilon_{0}\;\frac{\varepsilon_{r}+1}{2}\;\frac{K(k)}{K(k^{\prime })},\;k=\sin\;\left(\frac{\pi }{2}\;\frac{w}{w+s}\right),\;k^{\prime }=\sqrt{1-k^{2}},$$

is used (*K*: complete elliptic integral of the first kind; air and wood contribute one half each). The moisture dependence enters through the transverse permittivity of beech tabulated for a few kilohertz ([16], Chapter 5), $\varepsilon_{r}$ = 3.5, 4.2, 5.5, 7.5, 10.5, and 15.0 at 0, 6, 12, 18, 24, and 30% MC (Table S1). Such values depend on origin, density, frequency, and method; an uncertainty of ±20% is assumed; this is an estimate, not a confidence interval, of the order of two standard deviations of the density within a species (coefficient of variation about 10% [19]), on which the permittivity depends, plus differences in frequency and measurement method. As $\varepsilon^{\prime }$ of moist wood decreases with frequency, the model values at high MC are upper estimates for the 100 kHz readout. The model contains only the real part of the permittivity, i.e., no conductivity and no interfacial polarization; it describes the geometric and electrostatic behavior of the sensor but is not an impedance model of it at high MC (Section 3.2).

### 2.3. Design Space and Sensor Layout

The printable design space follows from $w_{\min}$: *w* from 210 μm to about $3\;w_{\min}$; *s* from 210 μm upward. The spacing controls the penetration depth, which is proportional to the spatial wavelength λ=2(w+s) [17]; the rule of thumb $d_{p}\approx \lambda /3$ served as a first estimate. The depth distribution was then computed from the field energy of the unit cell (finite-volume solution, Section 2.2; Figure S1a): for the base layout, 50%, 90%, and 99% of the wood-side field energy lies within 0.05, 0.20, and 0.41 mm of the surface, so λ/3 corresponds to the 99% depth, and a 10% change of $\varepsilon_{r}$ confined to the top 0.1 mm produces 66% of the full-substrate response. These depths are referred to as energy-based sensitivity depths; they give the share of the response for a uniform change of $\varepsilon_{r}$, whereas with a moisture gradient the depth from which the signal originates also depends on the local change of $\varepsilon_{r}$. The usable printing area limits *l* and the comb width to about 18 mm, so a larger spacing means fewer fingers and a lower base capacitance. The base layout is w=s=300 μm, N=12 finger pairs (24 fingers), and l=15 mm (Figure 1a): the width keeps a 40% margin above $w_{\min}$, the base capacitance of about 7 pF turned out to be about ten times the offset of the wiring determined later (0.69 pF at 100 kHz, Section 3.2), and the 99% depth of 0.4 mm covers the sanded layer. The comb (24w+23s=14.1 mm) is narrower than the usable 18 mm because N=12 was chosen as a safely printable reference point in the middle of the design window, from which *w* and *s* were varied [15]. Thirty fingers would give 9.0 pF instead of 7.2 pF (Figure S1b); as all capacitances scale with n−1, this would raise the base capacitance and the absolute sensitivity by about 25% but leave the relative response unchanged.

### 2.4. Impedance Measurement and Climate Protocol

Each sample was measured with its own inductance–capacitance–resistance (LCR) meter (two HM8118, HAMEG Instruments/Rohde & Schwarz, Mainhausen, Germany, read at common time stamps) in the parallel equivalent circuit $C_{p}\|R_{p}$ (test voltage 1 V, FAST mode, averaging mode MED: of six readings, the smallest and the largest are discarded and the remaining four averaged); the dissipation factor of the sensor is $\tan\delta =1/(\omega R_{p}C_{p})$; it is smaller than $\varepsilon^{\prime \prime }/\varepsilon^{\prime }$ of the wood because air and leads contribute lossless capacitance. As polarization effects caused by ionic conduction raise the apparent permittivity of moist wood at low frequencies [16], 1, 5, 20, and 100 kHz were recorded. The combs were contacted by polytetrafluoroethylene-insulated stranded wires (length 10 mm) soldered (solder paste CR11, Sn42Bi58, melting point 138 °C, Edsyn, Van Nuys, CA, USA) to the contact pads and connected with alligator clips, where the open/short compensation was performed, so the capacitance of the wires remains in all raw readings. The leads were strain-relieved, and the clips were left unchanged for the whole campaign. An identical structure on a 100 mm (4 in) borosilicate glass wafer (Borofloat 33, Schott, Mainz, Germany; $\varepsilon_{r}=4.6$; thickness 1.1 mm) with the same wiring served as reference for drift and instrumental effects (Figure 1d). The parallel representation is not an approximation at a given frequency: the meter determines the complex impedance, i.e., two quantities, which $C_{p}$ and $R_{p}$ represent exactly, as do the magnitude and phase of the impedance (Table S7). It is the natural equivalent circuit of a lossy dielectric, in which the displacement current through $C_{p}$ and the conduction and loss current through $R_{p}$ flow in parallel, and the usual choice for small capacitances with impedances far above 10 kΩ (here 85 kΩ to 23 MΩ). It is also the representation in which the capacitance depends on $\varepsilon^{\prime }$ alone: applying the linear relation of Section 3.1 to the complex permittivity gives $C_{p}=1.79\;pF+1.53\;pF\;\cdot \;\varepsilon^{\prime }$ and $G_{p}=1/R_{p}=\omega \;\cdot \;1.53\;pF\;\cdot \;\varepsilon^{\prime \prime }$ for the homogeneous model, so $C_{p}$ is the quantity the electrostatic model predicts. In the series representation, the same impedance would give $C_{s}=C_{p}\left(1+\tan^{2}\delta \right)$, e.g., 20.0 instead of 17.4 pF at 100 kHz in stage A90. Series elements are negligible: the resistance of a finger is at most 0.8 Ω ($R_{□}$ times 50 squares), which changes tanδ at 100 kHz by about $10^{-5}$, and even for an assumed upper bound of 1 μH for leads, clips, and test cable, the self-resonance with 10 to 30 pF lies above 25 MHz and changes the 100 kHz reading by less than $2\times 10^{-5}$. As polarization effects make $C_{p}$ and $R_{p}$ themselves frequency-dependent, no single circuit with constant elements describes all four frequencies. In the wet stages (tanδ>1 at 1 and 5 kHz in A90 and at 1 kHz in D70; Table S7), the current flows mainly through $R_{p}$ at low frequencies, whereas it flows mainly through $C_{p}$ at 100 kHz in all stages.

Both samples were conditioned in a climate chamber (C-40/200, CTS Clima Temperatur Systeme, Hechingen, Germany) at (23.00±0.07) °C (mean ± standard deviation; single readings of 22.7 to 26.2 °C at set-point changes) with the planned program 30% → 50 → 70 → 90 → 70 → 50 → 30% relative humidity (RH), 48 h per stage, and a final 100 h at 50% RH (sequence actually run: Table S2). As a deviation from this protocol, a programming error of the chamber control replaced the first adsorption stage at 50% RH by a ramp from 30 to 58% RH (Section 3.2). $C_{p}$ and $R_{p}$ were logged every 10 min. After a set-point change, whose exact time within the sampling interval is not recorded, the chamber RH was within 1% RH of the target from the third reading on, i.e., 20 min after the first reading and up to 30 min after the change; the second reading was still 0.9 to 3.9% RH off (Figure S2a). For the transverse diffusion coefficients measured for beech, D≈ 0.1 × 10 ^−10^–0.6 × 10 ^−10^ m^2^/s at 20 to 23 °C [20,21], the slowest diffusion time constant of the 15 mm disk exposed on both faces is $\tau_{1}=d^{2}/\left(\pi^{2}D\right)\approx$ 110–630 h, so the 48 h stages equilibrate a surface-near layer but not the disk. As gravimetric MC determination inside the running chamber was not feasible, the MC of each stage was assigned from the equilibrium moisture content (EMC) table of the Wood Handbook [19] (Table 4-2; Sitka spruce, midway between adsorption and desorption), linearly interpolated between 21.1 and 26.7 °C to 23 °C: 6.2%, 9.2%, 13.0%, and 20.4% MC at 30%, 50%, 70%, and 90% RH, the same for both branches. This table was derived primarily for Sitka spruce; according to the same source, EMC differences among species at room temperature are generally minor at low and moderate RH but become considerable at high RH [19,22]. The assignment is therefore least certain at 90% RH, the upper end of the MC span, where an error of one (two) percentage points changes the MC-referred sensitivity by 7 (14)% (Section 4); this uncertainty is not included in the ranges given for the MC-referred sensitivity. As no gravimetric calibration was performed, the MC values are estimates, referred to as assigned MC below; all MC values and MC-referred quantities in this paper rest on this assignment, and the RH-referred response is the primary experimental result. The conditioning stage at 30% RH was approached by desorption from the laboratory climate. The end value of a stage is the mean of its readings in the last 6 h before the next set-point change (35 per frequency). The readings were corrected with a calibration of the meters (main evaluation at 100 kHz; see below and Section 3.2). Step responses were fitted with $\left(C\left(t\right)-C_{\mathrm{end}}\right)/\left(C_{0}-C_{\mathrm{end}}\right)=a\;e^{-t/\tau_{s}}+\left(1-a\right)\;e^{-t/\tau_{l}}$ with *a*, $\tau_{s}$, and $\tau_{l}$ free, $C_{0}$ the mean of the 2 h before the step, and $C_{\mathrm{end}}$ the stage end value (95% confidence intervals from the covariance; single exponential for comparison by the Akaike information criterion, AIC). The times $t_{63}$ and $t_{90}$ at which 63% and 90% of the step are completed were read from 5-point median-filtered data.

Three weeks after the campaign, the measurement chain was calibrated with the settings of the campaign (FAST mode). C0G capacitors of 4.7, 10, and 22 pF (±0.1 pF), contacted with the same wiring as the samples, were measured on both LCR meters at the four frequencies, and a straight line, reading $=a+b\;C_{\mathrm{nom}}$, was fitted for each meter and frequency; calibrated values follow as $(C_{p}-a)/b$. The glass reference and the beech sensor were measured again in the same session (Table S3), in the laboratory climate; the laboratory temperature and RH during this session were not recorded; the C0G capacitors and the glass reference are largely insensitive to the moderate laboratory climate, and the beech readings of this session serve only to check the frequency behavior after calibration. The calibration thus describes the measurement chain at the time of this session; its transfer to the campaign data assumes an unchanged gain of the meter of the beech sensor (Section 3.2).

## 3. Results

### 3.1. Model Verification and Design Map

Three checks were made on the base layout at $\varepsilon_{r}=3.5$. Refining the edge length at the electrodes from 100 μm to about 11.5 μm without adaptive refinement changed $C_{\mathrm{cell}}^{\prime }$ from 21.00 to 20.59 pF/m (about 2%; 0.9% between the two finest levels). The unit cell (20.73 pF/m, adaptive) scaled to 23 gaps gives 476.90 pF/m against 486.21 pF/m for the full 24-finger comb (2D), an underestimate of 1.9% by the outer fingers. For w=s, Equation (2) gives $2.25\;\varepsilon_{0}=19.92\;pF/m$; the FEM values lie 3–4% above it, the contribution of the finite electrode thickness (1.5–4.9% over the design map). Finally, the simulated moisture response (Table S1) follows $C=1.79\;pF+1.53\;pF\;\cdot \;\varepsilon_{r}$ within 0.3%; the slope equals the analytical $\left(n-1\right)\;l\;\varepsilon_{0}/2=1.53\;pF$ per unit permittivity.

Figure 2a shows $C_{\mathrm{cell}}^{\prime }$ at $\varepsilon_{r}=3.5$ for four finger widths (210, 300, 450, and 630 μm) and five spacings (210, 400, 800, 1500, and 2000 μm). It ranges from 9.73 to 30.25 pF/m. At the full usable width of 18 mm, with n=⌊(18 mm+s)/(w+s)⌋ fingers and l=15 mm, this gives 1.1–13 pF (Figure S1b); 24 fingers fit into this width for only 5 of the 20 layouts. Against λ/3, which spans 0.28 to 1.75 mm in this map (Figure 2b), every curve falls monotonically: a deeper reach into the wood always costs base capacitance. For the largest spacing at the minimum width (w=210 μm, s=2000 μm), half of the wood-side field energy lies within 0.12 mm and 99% within 1.4 mm (Figure S1a). Field plots are given in Figure S3. For the base layout, *C* rises from 7.15 pF in the oven-dry state to 24.69 pF at fiber saturation (30% MC), by about 245%, convex like $\varepsilon_{r}\left(\mathrm{MC}\right)$; at 6.2% MC, the EMC assigned to 30% RH, the model predicts 8.26 pF.

### 3.2. Fabricated Sensor, Signal Attribution, Calibration, and Readout Frequency

The layout was fabricated with 24 continuous, non-merged fingers (Figure 1c). Figure 3 shows the 358 h campaign, which deviates from the plan (Table S2). The conditioning stage was a desorption step. Owing to a programming error, the first step was run as a ramp of 0.42% RH per hour from 30 to 58% RH, so both branches share only the 70% RH stage, and the final stage lasted only 3.7 h. From 174 h, the chamber reported a humidifier maintenance warning without a detectable deviation of the set values.

The beech capacitance follows every humidity change monotonically, rising by 97% at 100 kHz (calibrated) and by more than 300% at 1 kHz (raw). The glass reference, in contrast, changes by only 0.3–0.5% over the campaign, with a single step of 0.03 pF at t≈20 h of unknown cause (Figure 3c), so the signal is attributable to the wood. During the 90% RH stage the reference rises by up to 3.2% (1 kHz) and 2.3% (100 kHz) above its value in the first 6 h and decays afterwards, indicating a water film on the glass or the leads (0.2 pF).

The glass reference reveals an instrumental contribution but does not quantify it for the beech channel. During conditioning it reads 8.40, 8.51, 8.49, and 9.32 pF at 1, 5, 20, and 100 kHz; since glass shows no dispersion in this range, the rise of 0.92 pF between 1 and 100 kHz is instrumental. The dry beech reading rises even more (Table 1): by 1.64 pF (22%) between 20 and 100 kHz against 0.83 pF (10%) for the glass, and by 0.70 pF between 1 and 5 kHz (6.87 to 7.57 pF) against 0.11 pF for the glass. A capacitance increasing with frequency is impossible for a passive dielectric. The calibration explains the larger part of this discrepancy (Table S3). At 1, 5, and 20 kHz, both meters reproduce the reference capacitors with a gain *b* of 0.99 to 1.01 and an offset *a* of 0.48 to 0.73 pF (wires and pads); at 100 kHz, the gain is 1.218 for the meter of the beech sensor and 1.232 for that of the glass reference (*a* = 0.69 and 0.70 pF); no reference capacitor deviates from its line by more than 0.07 pF. Re-measured in the same session, the glass reference rises by 21 to 24% between 20 and 100 kHz and is flat after calibration (8.53–8.73 pF at all frequencies on both meters). Its mean of 8.69 and 8.61 pF on the two meters lies 1.4% and 2.4% below the model value of 8.81 pF for $\varepsilon_{r}=4.6$ (linear relation of Section 3.1; the 1.1 mm wafer acts as a half-space within 0.01%), an independent check of the model with a dielectric of known permittivity in the state of the calibration. During the campaign, the meter of the glass reference apparently operated with a smaller gain at 100 kHz (rise of 10%; implied gain 1.09), whereas the beech channel shows a gain of at least the calibrated size: after removal of the offsets, its dry 100 kHz reading exceeds the 20 kHz reading by a factor of 1.25, which for a passive dielectric is a lower bound of its gain during the campaign. The 100 kHz values of the beech sensor were therefore calibrated with the line of its own meter, $C=(C_{p}-0.69\;pF)/1.218$ (Table 1). The glass-referenced corrections (subtracting the rise of 0.92 pF of the glass reading between 1 and 100 kHz or dividing by the corresponding raw ratio of 1.109, as distinct from the offset-corrected gain of 1.09 above) would leave most of the gain error in the data (dry value 8.26 or 8.27 pF instead of 6.97 pF). As the gain of the glass meter changed between campaign and calibration, the calibration of the beech meter rests on the assumption that its gain did not; the campaign data imply a gain of at least 1.25 with the offsets of the calibration (at least 1.22 with offsets shifted as observed for the glass meter), so the calibration may under-correct by up to a few percent (2.3% for the bound of 1.246 with the offsets of the calibration, about 0.4% with shifted offsets; slightly more if $\varepsilon^{\prime }$ of the dry wood still decreases between 20 and 100 kHz), which would lower the sensitivities by the same fraction. The offsets during the campaign cannot be verified either: the glass readings at 1 to 20 kHz were 0.68 to 0.73 pF lower during the campaign than at calibration, and the campaign glass values converted with the calibration (7.81 to 7.91 pF) lie 10 to 11% below the model. If the offset of the beech meter shifted in the same way, its calibrated values would be up to 0.58 pF higher. The calibrated absolute values are therefore uncertain by −0.1/+0.6 pF (calibration fit and capacitor tolerance; possible offset shift; uncertainty budget: Table S4). The rise of the dry beech reading between 1 and 5 kHz is not explained by the calibration.

Three levels are therefore distinguished: (i) the raw campaign data (Table 1 and Table S7), which do not depend on the calibration; (ii) the agreement of the glass reference with the model, which applies only to the calibration session and is not a validation of the campaign; and (iii) the calibrated campaign values of the beech sensor, a retroactive correction that assumes an unchanged gain of its meter. A gain error scales the RH- and MC-referred sensitivities (by a few percent, see above), whereas a constant offset cancels in them and affects only the absolute values and thus the comparison with the model (Section 3.3); an offset that changes during the campaign, such as the water film on the leads in the 90% RH stage (see above), would not cancel; estimated from the glass reference, its effect is about 0.2 pF, i.e., up to about 3% of the change between 30 and 90% RH.

Table 1 lists the stage end values at all frequencies (plotted in Figure S4). At 1 kHz, the dissipation factor rises from 0.03 (30% RH) to 7.3 (90% RH). With tanδ≫1, the displayed parallel capacitance is no longer purely dielectric. As it then depends on the equivalent circuit used by the meter, the full impedance of all stage end values ($R_{p}$, conductance $G_{p}=1/R_{p}$, |Z|, and phase) is given in Table S7; in the series representation, the same impedance would give $C_{s}=C_{p}\left(1+\tan^{2}\delta \right)\approx 1.6\;nF$ instead of 29.4 pF at 1 kHz in A90, and at such loss factors small phase errors of the meter also enter $C_{p}$ strongly (at a phase of −8°, an error of 0.1° changes $C_{p}$ by about 1.3%). In the wet stage A90, $G_{p}$ changes by a factor of only 1.4 between 1 and 20 kHz (1.34 to 1.90 μS), whereas $C_{p}$ falls from 29.4 to 17.8 pF and the phase at 1 kHz is −8°: the low-frequency response is dominated by frequency-independent (dc-like) ionic conduction, which enters only $G_{p}$, and by the polarization it causes at interfaces and at the bare silver electrodes, which raises $C_{p}$. At 90% RH, 29.4 pF is read at 1 kHz but 17.4 pF at 100 kHz (raw, −41%), whereas the dry reading increases with frequency. At 100 kHz, tanδ≤0.39. The glass reference has a mean tanδ≤0.012 at all four frequencies during conditioning (single readings ≤ 0.013, at most 0.019 in the wet stages). The dry beech shows 0.03, 0.02, and 0.09 at 1, 20, and 100 kHz; as the reference capacitors and the glass reference showed 0.03 and 0.07 at 100 kHz in the calibration session, the rise is largely instrumental. At 20 kHz, the meters show no gain error and the sensitivity is higher (0.17 against 0.11 pF per percentage point of RH between 30% and 90% RH, both calibrated), but tanδ reaches 0.85 at 90% RH. The evaluation therefore uses 100 kHz, with the smallest dissipation factor in the wet state. Even there, tanδ=0.39 at 90% RH, so $C_{p}$ at high RH remains an apparent capacitance. Between 20 and 100 kHz, the calibrated A90 value still falls from 17.1 to 13.7 pF, whereas the dry value rises by 2.3%; the RH-referred sensitivity thus decreases from 0.17 to 0.11 pF per percentage point. The reported sensitivity is therefore specific to the 100 kHz readout and may still contain a contribution of conduction-related (interfacial and electrode) polarization, which would decrease further at higher frequencies.

### 3.3. Static Transfer Characteristic and Model Comparison

On the adsorption branch, the calibrated capacitance rises from 6.97 to 13.70 pF between 30% and 90% RH (+97%). This corresponds to 0.112 pF per percentage point of RH (1.61% of the base capacitance) or 0.47 pF per percentage point of assigned MC (0.46 pF for a gain of 1.25, Section 3.2). As the tabulated EMC lies midway between the branches, the desorption-approached start lies above and the A90 point below the assigned MC; for a branch difference of 1 (2) percentage points [23], the span shrinks accordingly and the value becomes 0.51 (0.55) pF. The MC-referred sensitivity is thus 0.46–0.55 pF per percentage point (gain and a branch difference of 1 to 2 percentage points), excluding the uncertainty of the tabulated EMC for beech (Section 2.4). The transfer characteristic is markedly nonlinear. The local sensitivity grows from 0.047 (30 to 70% RH) to 0.24 pF per percentage point of RH (70 to 90% RH), or from 0.28 to 0.66 pF per percentage point of assigned MC, a factor of 2.4 against 1.65 in the model (0.36 to 0.59 pF).

In the last 16 h of conditioning, the standard deviation of a single raw 100 kHz reading is 66 fF, against 3 fF for the glass channel. The beech readings are white and uncorrelated with chamber RH and temperature (Table S4); the cause (meter, contact, or lossy wood) is open. The running mean of six readings (1 h) scatters by 29 fF. After division by the gain, the 3σ values of the 1 h mean and of a single reading are 71 and 164 fF. Under the present measurement conditions, this gives a short-term, noise-limited 3σ resolution of 0.6–1.5% RH with the average sensitivity and 1.5–3.5% RH with the dry-range local sensitivity (0.047 pF per percentage point of RH), each from the 1 h mean to the single reading, or 0.15–0.35 and 0.26–0.59 percentage points of assigned MC (0.47 and 0.28 pF per percentage point). This resolution is not the accuracy of the sensor: it does not include the calibration uncertainty, the MC assignment, or hysteresis. The accuracy is dominated by the MC assignment, the hysteresis bound, and the return offset.

The calibrated characteristic has the convex shape of the model and its average sensitivity (Figure 4a): over 6.2 to 20.4% MC, the model predicts 0.48 pF per percentage point against the measured 0.46 to 0.47 (0.51 for a branch difference of 1 percentage point). The absolute values, however, lie below the model: with the offsets of the calibration, the adsorption points deviate by −15.7% at 6.2% MC, −17.2% at 13.0% MC, and −9.1% at 20.4% MC from the model values 8.26, 10.71, and 15.07 pF (linear fit $C(\varepsilon_{r})$ with $\varepsilon_{r}$ interpolated in Table S1), i.e., by 1.3 to 1.8 pF; with an offset shifted as observed for the glass meter, by −8.6%, −11.8%, and −5.2%. The point at 6.2% MC lies at the lower edge of the band of the assumed ±20% permittivity uncertainty, the point at 13.0% MC just below it, and the point at 20.4% MC inside it; the desorption points lie 2 to 13% below the model (Section 3.4). The deviation thus persists within the offset uncertainty: even with an offset shifted as observed for the glass meter, the adsorption points remain 5 to 12% below the model. It is therefore more likely a property of the probed wood layer than an instrumental offset, although an instrumental share cannot be excluded, as the glass reference itself read 10 to 11% below the model during the campaign (Section 4). The stronger curvature (factor 2.4 against 1.65) is a genuine deviation of shape; candidates are conduction-related (interfacial and electrode) polarization still present at tanδ=0.39, the water film on the leads, and an MC of the treated surface layer at 90% RH above the assigned value. The comparison with the electrostatic model is therefore limited to the geometric and electrostatic trend. Interfacial and electrode polarization, both caused by ionic conduction, raise the measured apparent capacitance (a frequency-independent conductance enters only $G_{p}$), and the kilohertz permittivities raise the model values at 100 kHz; both effects grow with MC and act in opposite directions on the upper end of the characteristic, i.e., mainly on the local sensitivity between 70% and 90% RH. The agreement of the average sensitivity (0.47 against 0.48 pF per percentage point) therefore does not by itself validate the model at high MC. Converted with $C(\varepsilon_{r})$, the values give effective permittivities of 3.4, 4.6, and 7.8 against the tabulated 4.2, 5.8, and 8.7 (Figure S5; not an independent test).

### 3.4. Dynamic Response, Hysteresis, and Reversibility

The five fully recorded step responses (Figure 4b; logarithmic time: Figure S6) show two separated time scales. The two-phase model describes them with residuals of 73–113 fF (raw readings), whereas a single exponential leaves 117–529 fF; the residuals of the two-phase fit are autocorrelated (lag-1 coefficient up to 0.52), which inflates the ΔAIC of 240 to 880 in its favor and makes the covariance-based confidence intervals too narrow, by up to a factor of about 1.8 (variance inflation $\sqrt{\left(1+r_{1}\right)/\left(1-r_{1}\right)}$ for first-order autocorrelation; Table S5). Between 47% and 66% of every change (a=0.47 to 0.66) occurs with $\tau_{s}$ = 0.47–0.80 h for adsorption. For desorption, the fit gives 0.22–0.34 h (13–20 min), comparable to the chamber settling time and the sampling interval, so the fast desorption response is not resolved (≲0.5 h). The time origin is uncertain by up to 10 min, and the first reading after a set-point change already contains 6 to 13% of the step (Figure S2b). The second part proceeds with $\tau_{l}$ = 9.7–13.3 h. The response is asymmetric, $t_{90}$ = 10.7–13.2 h for desorption against 15.4–18.7 h for adsorption, which is consistent with a diffusion coefficient increasing with MC [24] and with desorption starting from the wetter state. The two-phase model is therefore used as an empirical description of the dynamics, not as the identification of two distinct physical mechanisms: the fast desorption component is not resolved, the time origin is uncertain by up to 10 min, and the confidence intervals are too narrow because of the autocorrelated residuals. Even for cell-wall sorption alone, the parameters of two-exponential (parallel exponential kinetics) fits have been shown not to be physically meaningful [25].

At 70% RH, the only RH level held on both branches, the desorption value exceeds the adsorption value by 1.61 pF (18%; calibrated) at 100 kHz but by 6.8 pF (65% calibrated with the 1 kHz line of Table S3, 61% raw) at 1 kHz, largely an after-effect of the conduction-related (interfacial and electrode) polarization of the preceding wet stage (Figure S7). With the average sensitivity of 0.47 pF per percentage point, 1.61 pF corresponds to 3.4 percentage points of assigned MC (2.5 to 5.8 with the local sensitivities above and below 70% RH). This is an upper bound of the sensor hysteresis at 70% RH only; with a single RH level held on both branches, the hysteresis loop itself is not characterized. The same midway EMC was assigned to both branches, whereas the desorption EMC of wood is typically one to two percentage points above the adsorption EMC [23], which also explains why the desorption points in Figure 4a lie above the adsorption points. In addition, the raw 100 kHz reading still changed by 0.4–2.0% during the last 18 h of each stage, so adsorption end values underestimate and desorption end values overestimate the equilibrium. After the closed cycle 30 → 90 → 30% RH (both 30% RH states approached by desorption), the calibrated capacitance is 7.15 pF, 0.19 pF or 2.7% above the initial value. This corresponds to 0.4 to 0.7 percentage points of assigned MC with the average or the dry-range sensitivity; an irreversible change of the printed structure is not detectable within this bound.

## 4. Discussion

Equation (2) reproduces the design map within 5%; the FEM adds the electrode thickness and the basis for layered or 3D extensions. The model assumes a homogeneous moisture distribution and contains no conductivity or interfacial polarization. It is two-dimensional and neglects the finger ends facing the opposite bus bar (about +2%, estimated as 24w/(23l)), the bus bars, and the pads. It represents the printed lines as rectangles, although they are lens-shaped and partly embedded in the porous surface. On glass, with a smooth surface and a known permittivity, the reading measured together with the calibration lies 1.4 to 2.4% below the two-dimensional model, or 5 to 6% below it if the estimated end effects (+4%: outer fingers and finger ends) are included; the model, corrected for the estimated end effects, thus agrees with the glass reference within about 6%. On beech, the average sensitivity agrees with the model, whereas the absolute values on the adsorption branch lie 5 to 17% below it (1.3 to 1.8 pF with the offsets of the calibration); i.e., the effective permittivity is lower than tabulated by 0.8 to 1.2 on this branch (Figure S5). A probed layer with a lower permittivity than the tabulated solid beech is plausible: two thirds of the response stems from the top 0.1 mm, a layer that is sanded, heated to 150 °C during curing, and partly covered by silver, and the tabulated values themselves depend on density and are uncertain (Section 2.2). The heated layer may also follow an isotherm different from the tabulated one; a lower MC than assigned would act in the same direction. An instrumental share remains possible: converted with the same calibration, the glass reference read 10 to 11% below the model during the campaign, so the offsets then differed from those at calibration. Calibrating before and after each campaign, or a reference capacitor on a separate channel, would remove this uncertainty.

The sensor probes mainly the near-surface wood (90% of the sensitivity within 0.2 mm, two thirds of the response from the top 0.1 mm; Section 2.3), a layer that is sanded, heated during curing, and partly covered by silver; the fast fraction is consistent with the response of this layer. The slow fraction is not the response of the disk, whose time constant $\tau_{1}\approx$ 110–630 h (Section 2.4) exceeds $\tau_{l}$ by an order of magnitude. Conversely, diffusion alone would bring the probed layer to 90% of a surface step within 1–6 h (semi-infinite solution with the beech values of *D*, weighted with the energy distribution of Figure S1a). The slow fraction therefore may point to a resistance at the surface or in the cell wall, i.e., a surface emission resistance, the slow component of cell-wall sorption (parallel exponential kinetics [26]), or the vapor barrier of the fingers, and possibly to lateral exchange through the unsealed rim, where end grain is exposed. The present experiment cannot distinguish these candidates; a layered, diffusion-coupled model with surface resistance, together with experiments that vary the exposed area and the electrode coverage, is needed to separate these contributions.

Table S6 compiles related sensors. The paper IDC of [12] gave about 2 pF per percentage point of RH at 100 kHz over 40–100% RH, an absolute value for a thin, fully probed substrate (here 0.11 pF or 1.6% of the base capacitance). For pencil-drawn graphite IDCs on pine, a relative change rising from about 0.1 to 0.65 between 30 and 90% RH was reported [11] (reference state not stated), whereas the calibrated capacitance here rises by 97% over this range.

A denser comb raises the capacitance per area and thus the sensitivity relative to the fixed parasitic offset, but it shortens the probed depth in proportion to the pitch (Figure 2). Within the present line-width limit, the densest layout (w=s=210 μm, 43 fingers over 18 mm) would raise the base capacitance from 7.2 to about 13 pF (Figure S1b), and the absolute sensitivity in proportion, at the cost of a 90% depth reduced from 0.20 to about 0.14 mm and of no margin above $w_{\min}$. For a moisture sensor, the design is therefore a trade-off between signal and probed depth rather than a maximization of capacitance density. Using the full usable width with the present layout (30 instead of 24 fingers) raises the base capacitance and the absolute sensitivity by about 25% without this trade-off (Section 2.3); finer lines from smaller nozzles or other printing processes would extend the design space below $w_{\min}$; and electrodes embedded in the wood volume, for which the FEM model can be extended to layered or three-dimensional geometries, would probe deeper layers. For miniaturized designs on wood or other biomaterials, the FEM gives a simple scaling rule: for w=s, the 90% energy-based sensitivity depth is about one third of the pitch (0.20 mm at 600 μm). A comb with a pitch of 100 μm, as in inkjet-printed humidity sensors on foil [13], would thus probe only the top ∼30 μm, i.e., mainly the layer modified by sanding, curing, and the electrodes, at a depth comparable to the cell dimensions of wood, where the description by a homogeneous permittivity, on which the model rests, becomes questionable. For miniaturized biomaterial-based sensors, the characterization of this layer therefore becomes more important than the accuracy of the field solution. Combs of several pitches on the same sample, as in multi-wavelength sensors [5], would separate the permittivity of this layer from that of the bulk, analogous to the series of layer thicknesses with which the permittivity of nanometre-thin molecular films has been determined in rolled-up nanomembrane capacitors [27].

Limitations: The study is based on one sensor on one beech sample, so nothing can be said about the spread between sensors; the printability of the layout was demonstrated once. Variability from the wood (density, anisotropy, and heterogeneity), from the surface roughness and porosity, and from the DIW geometry cannot be separated, so all values describe the response of one device (proof of concept) and not the sensor technology. MC was not measured gravimetrically but assigned from EMC values tabulated for Sitka spruce, not for the beech used and its cured surface layer; an error of one percentage point at one or both ends of the 14.2-point MC span changes the MC-referred sensitivity by 7 or 14%, whereas the RH-referred characteristic, hysteresis, and dynamics are unaffected. Hysteresis and reversibility are upper bounds. The 100 kHz readings contain a gain error of 22% that was calibrated only three weeks after the campaign; the gain of the other meter changed in between, the campaign data imply a gain of the beech meter of at least 1.22 to 1.25, depending on the offsets, and the offset during the campaign is uncertain by −0.1/+0.6 pF. The sensor probes mainly the top 0.2 mm, whereas decay depends on the MC deeper in the member; the calibrated range ends at 20.4% MC, and liquid water was not tested. The silver electrodes are bare and exposed to sulfidation and silver migration; encapsulation, adhesion, durability, and the temperature cross-sensitivity were not investigated (the chamber was held at (23.00±0.07) °C, so temperature effects on the stage end values are negligible; the excursions to 22.7 to 26.2 °C at set-point changes coincide with the first readings of the step responses), and stability was assessed over 15 days only.

## 5. Conclusions

As a proof of concept with one sensor on one sample, an IDC was written directly onto beech wood by DIW of a silver paste. The geometry (w=s=300 μm, 24 fingers, *l* = 15 mm) was derived from a numerically verified electrostatic unit-cell model within the smallest printable line width of about 210 μm; 90% of its sensitivity lies within 0.2 mm of the surface, so it reports the moisture of the surface layer. A calibration with reference capacitors revealed a gain error of the meters of 22% at 100 kHz; after calibration, an identically printed glass reference measured in the same session agreed with the two-dimensional model within 3%, whereas its campaign readings lay 10 to 11% below the model, so the offsets during the campaign remain uncertain. At 100 kHz, the calibrated sensor response rose monotonically over a 15-day sorption cycle with 0.11 pF per percentage point of RH between 30% and 90% RH, or 0.47 pF per percentage point of assigned MC (0.46–0.55 pF including gain and sorption-branch uncertainty, excluding the species-related EMC uncertainty), close to the model value of 0.48 pF, with a short-term, noise-limited 3σ resolution under the present measurement conditions of 0.6–3.5% RH or 0.15–0.59% MC. As no gravimetric calibration was performed, the RH-referred value is the primary result and the MC-referred value an estimate; at high RH, the capacitance at 100 kHz still contains a contribution of conduction-related polarization (tanδ=0.39), so the electrostatic model describes the trend but not the full impedance. The absolute values lie 5 to 17% below the model, which points to a lower permittivity of the probed surface layer than tabulated, a lower MC than assigned, or a remaining instrumental offset. The step response is two-phase, with an unresolved fast fraction and a slow one of 10–13 h, and the sensor returned to within 2.7% of its initial value after the closed cycle. Future work should calibrate the measurement chain before and after each campaign and add several sensors with a gravimetric isotherm of the treated surface, temperature tests, encapsulation, long-term stability tests, broadband impedance spectra with an equivalent-circuit analysis, and a layered, diffusion-coupled model.

## Figures and Tables

**Figure 1:**
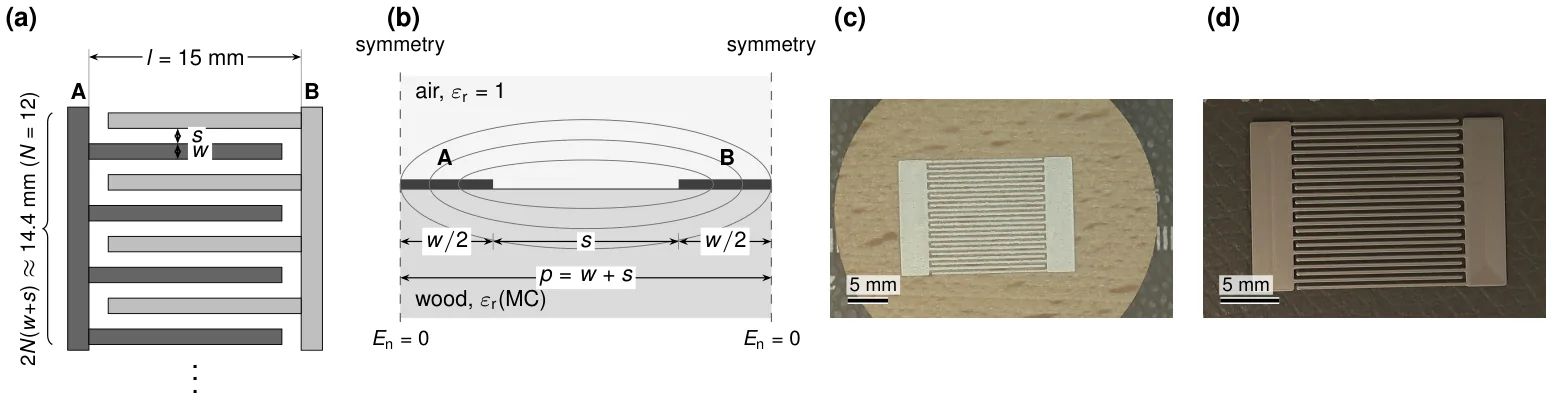
(**a**) Top view of the IDC with finger width *w*, spacing *s*, length *l*, and *N* finger pairs (base layout: w=s=300 μm, N=12, l=15 mm). (**b**) Periodic unit cell of the two-dimensional model with symmetry planes ($E_{n}=0$) through the finger centers. (**c**) Sensor printed by DIW on the radial surface of the beech sample; (**d**) identically printed reference on a glass wafer. Scale bars 5 mm, derived from the finger pitch of 600 μm.

**Figure 2:**
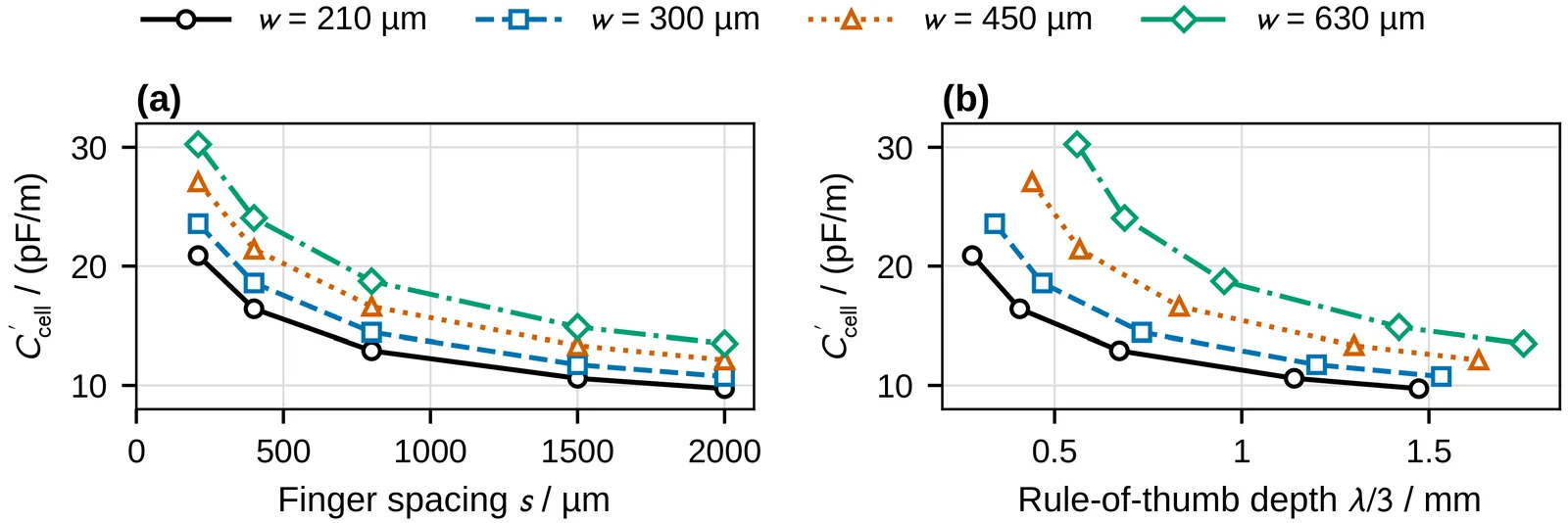
Simulated design map of the unit-cell capacitance per unit length $C_{\mathrm{cell}}^{\prime }$ at $\varepsilon_{r}=3.5$: (**a**) versus finger spacing *s* for four finger widths *w*; (**b**) versus the rule-of-thumb depth λ/3=2(w+s)/3, which corresponds to the 99% energy-based sensitivity depth (Figure S1a).

**Figure 3:**
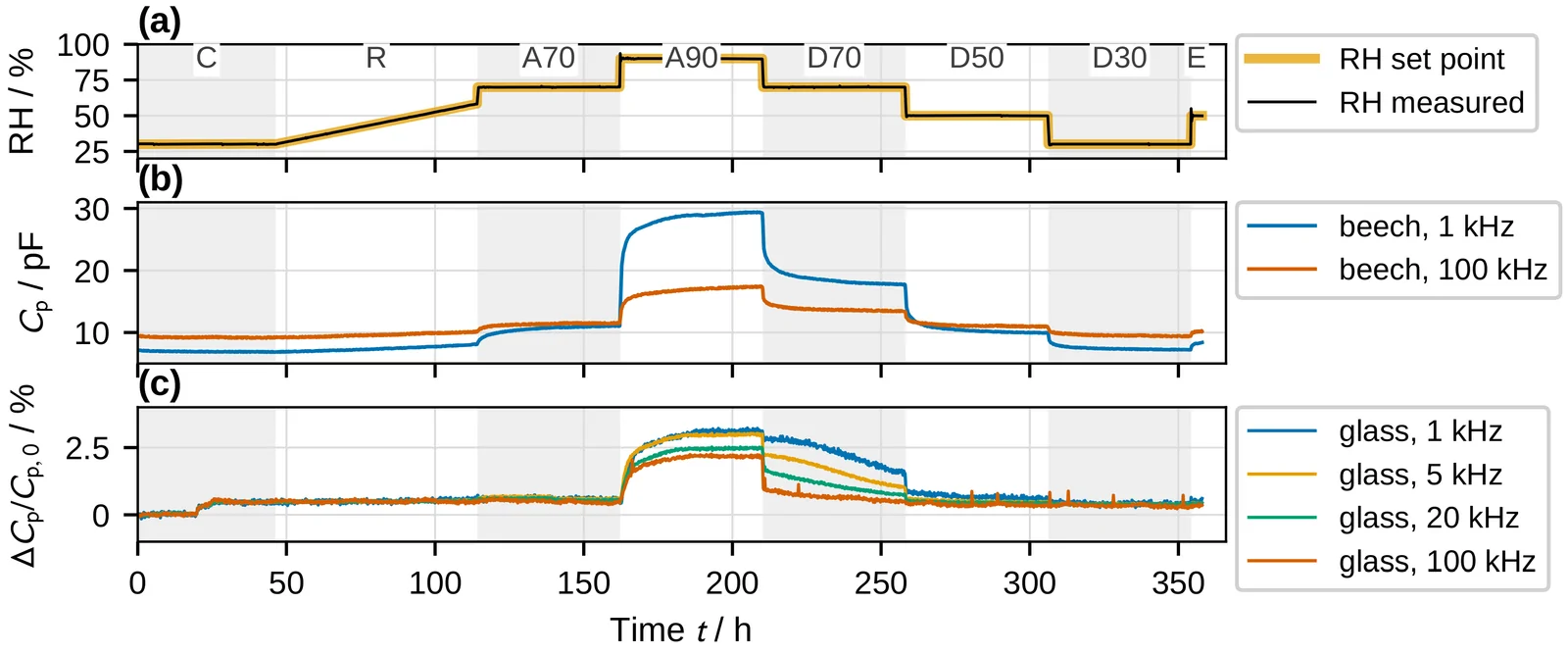
Sorption campaign at 23 °C: (**a**) set point (thick) and measured (thin) RH with the phase codes C (conditioning), R (ramp), A70/A90 (adsorption), D70/D50/D30 (desorption), and E (end); (**b**) capacitance of the beech sensor at 1 and 100 kHz (raw values); (**c**) relative change of the glass reference at the four frequencies.

**Figure 4:**
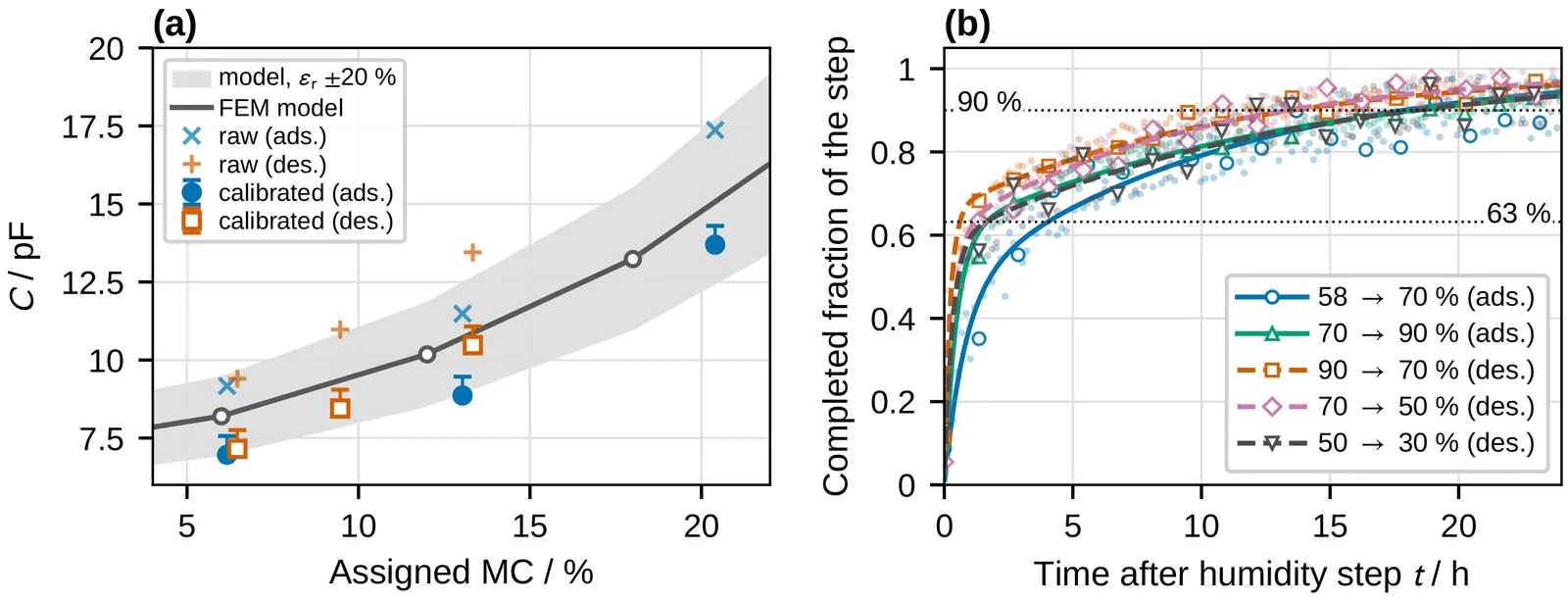
(**a**) Static transfer characteristic: raw and calibrated 100 kHz values (error bars −0.1/+0.6 pF, uncertainty of the calibrated values; desorption markers offset horizontally by +0.3% MC for visibility) over the assigned MC, with the simulated characteristic and the band of ±20% in $\varepsilon_{r}$ (assumed uncertainty of the tabulated permittivity used as model input, Section 2.2; causes of the deviation: Section 4). (**b**) Completed fraction of the step, $\left(C-C_{0}\right)/\left(C_{\mathrm{end}}-C_{0}\right)$ = 1 − the normalized expression of Section 2.4, at 100 kHz (t=0 at the first reading after the set-point change; points, hollow markers every eighth reading) with the fitted two-phase curves; solid: adsorption; dashed: desorption; dotted lines mark 63 and 90%.

**Table 1:** Stage end values of the beech sensor (mean of the last 6 h before the next set-point change): raw $C_{p}$ at the four frequencies, the calibrated 100 kHz value $C=(C_{p}-0.69\;pF)/1.218$ (Table S3; from unrounded values), and tanδ at 1 and 100 kHz; MC assigned from the EMC table [19] at 23 °C; C (des.): conditioning stage, approached by desorption.

Phase	RH (%)	MC (%)	$C_{p}$ (pF)				*C*, cal. (pF)	tanδ	
			1 kHz	5 kHz	20 kHz	100 kHz	100 kHz	1 kHz	100 kHz
C (des.)	30	6.2	6.87	7.57	7.53	9.17	6.97	0.030	0.092
A70 (ads.)	70	13.0	11.04	10.40	9.82	11.49	8.87	0.365	0.113
A90 (ads.)	90	20.4	29.36	22.86	17.81	17.37	13.70	7.26	0.393
D70 (des.)	70	13.0	17.79	14.25	12.26	13.45	10.48	1.47	0.176
D50 (des.)	50	9.2	9.96	9.71	9.28	10.98	8.45	0.265	0.106
D30 (des.)	30	6.2	7.24	7.85	7.77	9.40	7.15	0.050	0.095

## Data Availability

Raw campaign data, extracted simulation data, and the scripts generating all figures are available from the corresponding author on request.

## Supplementary Materials

The following supporting information can be downloaded at https://www.mdpi.com/article/10.3390/s26216968/s1: Figure S1: Depth distribution of the electrostatic field energy and total capacitance of the design-map layouts; Figure S2: Response in the first hours after the set-point changes; Figure S3: Magnitude of the electric field in the cross-section of the full 24-finger comb; Figure S4: Frequency dependence of the parallel capacitance and the dissipation factor of the beech sensor; Figure S5: Effective permittivity of the wood back-calculated from the calibrated 100 kHz values; Figure S6: Step responses on a logarithmic time axis; Figure S7: Hysteresis and non-reversibility as a function of the measurement frequency; Table S1: Simulated moisture response of the base layout; Table S2: Actual sequence of the sorption campaign; Table S3: Calibration of the measurement chain; Table S4: Uncertainty budget of the 100 kHz capacitance of the beech sensor and noise analysis; Table S5: Step responses of the beech sensor at 100 kHz; Table S6: Capacitive wood-moisture sensors, printed sensors on wood, and printed capacitive humidity sensors compared with this work; Table S7: Full impedance of the beech sensor at the stage end values. References [4,5,6,8,10,11,12,13,17,19,28] are cited in the supplementary materials.
