# Supplementary material for: Direct-Ink-Written Interdigital Capacitive Sensor on Beech Wood for Surface Moisture Monitoring: Electrostatic FEM Design and Proof-of-Concept Characterization
Source: Sensors (Basel). 2026 Nov 4;26(21):6968. doi: 10.3390/s26216968 (PMC13766497; doi:10.3390/s26216968)
Supplement: Supplementary file 1 [file sensors-26-06968-s001.zip › sensors-4635748-supplementary.pdf]

Supplementary Materials

Direct-Ink-Written Interdigital Capacitive Sensor on Beech Wood for Surface Moisture Monitoring: Electrostatic FEM Design and Proof-of-Concept Characterization

Paul Reber, Philipp Kersten, Sören Majcherek and Sören Hirsch

Department of Engineering, Technische Hochschule Brandenburg, Brandenburg an der Havel, Germany

The items are numbered in the order of their first citation in the main text: Table S1, Figure S1, Table S2, Figure S2, Table S3, Figure S3, Table S4, Figures S4–S6, Table S5, Figure S7, Table S6; Table S7, added in the revision, follows at the end.

Table S1: Simulated moisture response of the base layout ($w = s = 300\,\mu\text{m}$, 24 fingers/23 gaps, $l = 15\,\text{mm}$, electrode thickness 12 µm, comb 14.1 mm × 15 mm). ε_r: transverse permittivity of beech assigned to each MC (Torgovnikov, 1993; see the reference list of the main text); C'_cell: computed unit-cell value; $C = 23\,C'_\text{cell}\,l$. The values follow $C = 1.79\,\text{pF} + 1.53\,\text{pF} \cdot \varepsilon_\text{r}$ within 0.3 %; the model values quoted in the main text at the assigned MC of 6.2, 9.2, 13.0, and 20.4 % (8.26, 9.25, 10.71, and 15.07 pF) are obtained from this linear relation with ε_r interpolated linearly between the rows. The uncertainty of ±20 % assumed for the tabulated ε_r corresponds to ±1.3, ±1.8, and ±2.7 pF at 6.2, 13.0, and 20.4 % MC (band in Figure 4a of the main text). Sensitivity to the oven-dry value: with $\varepsilon_\text{r}(0\,\%) = 2.5$ instead of 3.5, the oven-dry capacitance would be 5.6 pF instead of 7.1 pF (−21 %; the design map of Figure 2, computed at $\varepsilon_\text{r} = 3.5$, scales with $(\varepsilon_\text{r} + 1)$, i.e., by 0.78), and the base capacitance would be eight instead of ten times the offset of the wiring (0.69 pF at 100 kHz, Table S3); the model values in the measured range (6.2–20.4 % MC, interpolated between the rows at 6 % MC and above) and the depth distribution of Figure S1a (thin electrodes) are unchanged.

MC (%)	ε_r	C'_cell (pF/m)	C (pF)
0	3.5	20.74	7.154
6	4.2	23.76	8.198
12	5.5	29.51	10.180
18	7.5	38.36	13.234
24	10.5	51.64	17.817
30	15.0	71.58	24.694

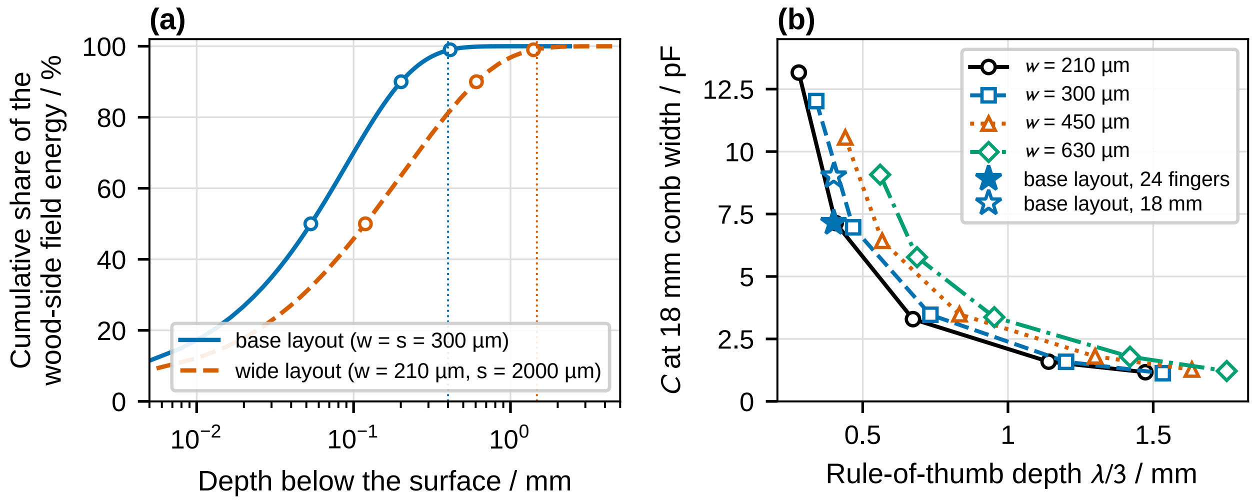

Figure S1: (**a**) Cumulative share of the wood-side electrostatic field energy within a given depth below the surface for the base layout ($w = s = 300\,\mu$m) and the layout with the largest spacing at the minimum width ($w = 210\,\mu$m, $s = 2000\,\mu$m), computed from a two-dimensional finite-volume solution of Equation (1) of the main text on the periodic unit cell with infinitely thin electrodes and a homogeneous substrate ($\varepsilon_r = 3.5$; the total C'_{cell} agrees with the analytical Equation (2) within 0.5 %). Circles mark the depths containing 50, 90, and 99 % of the energy (base layout 0.05, 0.20, and 0.41 mm; wide layout 0.12, 0.61, and 1.40 mm); dotted vertical lines mark the rule-of-thumb depth $\lambda/3$ (0.40 and 1.47 mm), which thus corresponds to the 99 % depth. The penetration depth is proportional to λ [S10]; the factor 1/3 is taken from this calculation. To first order the energy share equals the sensitivity share: a 10 % increase of ε_r confined to the top 0.05, 0.10, 0.20, and 0.40 mm of the base layout produces 44, 66, 89, and 99 % of the full-substrate response (two-layer calculation with the same solver). Script: `scripts/depth_sensitivity.py`. (**b**) Total capacitance of the 20 design-map layouts at the full usable comb width of 18 mm: $n = \lfloor (18\,\mathrm{mm} + s)/(w + s) \rfloor$ fingers, $C = (n-1)\,C'_{\mathrm{cell}}\,l$ with $l = 15$ mm and $\varepsilon_r = 3.5$ (1.1–13.2 pF); stars: base layout with 24 fingers (7.2 pF, 14.1 mm wide) and with 30 fingers at 18 mm (9.0 pF). With 24 fingers ($24w + 23s \leq 18$ mm) only 5 of the 20 layouts fit into the usable width. Script: `scripts/make_figs_paper.py`.

Table S2: Actual sequence of the sorption campaign at 23 °C: start and duration of each phase, mean measured relative humidity (RH) of the last 6 h, and the moisture content (MC) assigned from the equilibrium moisture content (EMC) table of the Wood Handbook ([S11], Table 4-2; Sitka spruce, midway between adsorption and desorption): 6.2/9.2/13.1/20.5 % at 21.1 °C and 6.1/9.1/12.9/20.2 % at 26.7 °C for 30/50/70/90 % RH, linearly interpolated to 23 °C; n/a: not assigned (no equilibrium). The master's thesis had used EMC nodes at 20 °C (4.5/6.8/9.3/12.0/16.0/20.5 % at 20/35/50/65/80/90 % RH, i.e., 6.0/9.3/13.3/20.5 % MC at the set points). The planned program was 30 % → 50 → 70 → 90 → 70 → 50 → 30 % RH with 48 h per stage and a final 100 h at 50 % RH. From 174 h the chamber reported a humidifier maintenance warning without any detectable deviation of the set values. The conditioning stage was approached by desorption from the laboratory climate.

Phase	Code	RH Set (%)	Start (h)	Duration (h)	RH Meas. (%)	MC (%)
Conditioning (des.)	C	30	0	46.3	30.0	6.2
Ramp	R	30 → 58	46	67.9	57.1	n/a
Adsorption	A70	70	114	48.0	70.0	13.0
Adsorption	A90	90	162	48.0	90.0	20.4
Desorption	D70	70	210	48.0	70.0	13.0
Desorption	D50	50	258	48.0	50.0	9.2
Desorption	D30	30	306	48.0	30.0	6.2
End	E	50	354	3.7	42.6	n/a

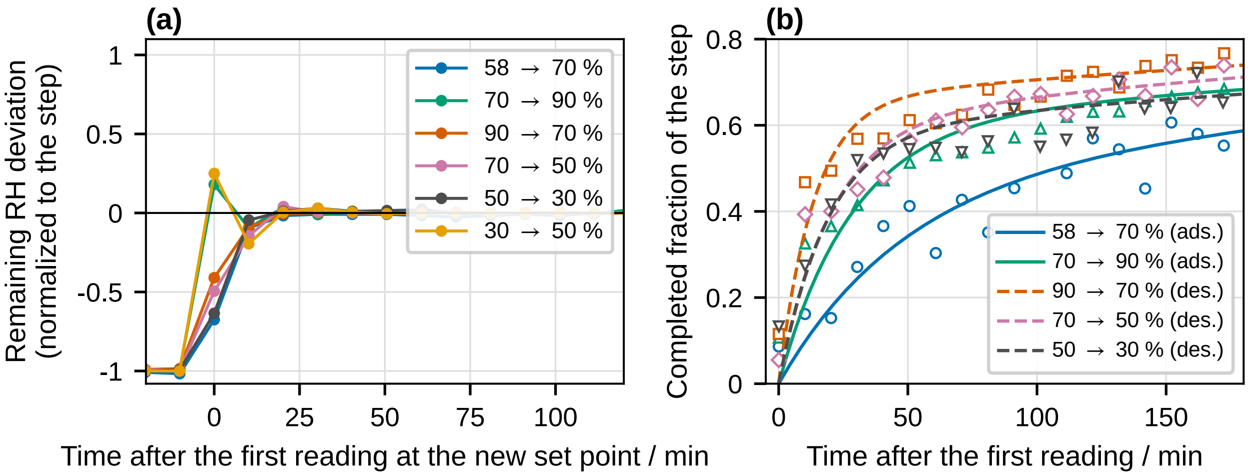

Figure S2: Response in the first hours after the set-point changes (10 min sampling). Time zero is the first reading at the new set point; the set-point change occurred between the last reading before and this reading, i.e., up to 10 min earlier (not recorded). (**a**) Remaining deviation of the measured chamber RH from the new set point, normalized to the step height. The first reading shows the RH still in transition or overshooting (by up to 5 % RH), the second reading deviates by 0.9 to 3.9 % RH, and from the third reading on (20 min, i.e., up to 30 min after the change) the deviation is within 1 % RH for all six steps. (**b**) Completed fraction of the step of the 100 kHz capacitance of the beech sensor in the first 3 h (markers) with the two-phase fits of Table S5 (fixed C_0 and C_{end}). The first reading already contains 6 to 13 % of the step; the fast fraction of the desorption steps (τ_s = 13–20 min from the fit) is resolved by only one or two readings, coincides with the chamber settling time, and is therefore reported as not resolved ($\lesssim$ 0.5 h).

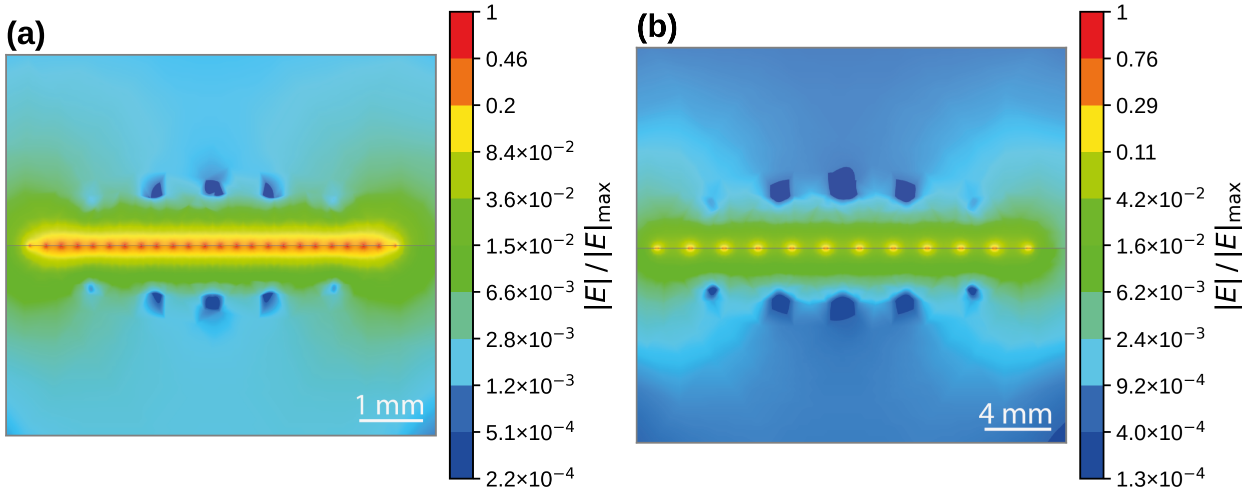

Figure S3: Magnitude of the electric field in the cross-section of the full 24-finger comb (two-dimensional electrostatic FEM model in Ansys Maxwell, arbitrary excitation, $\varepsilon_r = 3.5$), normalized to the maximum of each panel and shown on the logarithmic ten-band color scale of the solver: (**a**) base layout ($w = s = 300\,\mu\text{m}$) with a surface-near, strongly coupled field; (**b**) wide arrangement ($w = 210\,\mu\text{m}$, $s = 2000\,\mu\text{m}$) with deeper penetration but weaker coupling. Note the different scale bars (1 and 4 mm). The dark polygons far from the electrodes are regions below the lowest contour level ($|E|/|E|_{\max} < 3 \times 10^{-4}$) on the coarse outer mesh; their field energy is negligible for the capacitance. The field enhancement at the electrode edges is the reason for the adaptive mesh refinement there.

Table S3: Calibration of the measurement chain three weeks after the campaign (campaign 26 August to 10 September, calibration 2 October 2026; HM8118, 1.00 V, FAST mode, averaging MED, C–R mode, open/short compensation at the alligator clips, as in the campaign). C0G capacitors of 4.7, 10, and 22 pF (±0.1 pF), contacted with the same wiring as the samples, were measured on the meter of the beech sensor (LCR 1) and on that of the glass reference (LCR 2); a and b: straight-line fit, reading $= a + b\,C_{\text{nom}}$; max. res.: largest deviation of a capacitor from its line. Glass reference and beech sensor: readings of the same session, raw / calibrated, $(C_{\text{p}} - a)/b$ (beech in the laboratory climate; laboratory temperature and RH were not recorded). After calibration, the glass reference is flat over frequency (mean 8.69 and 8.61 pF; model value 8.81 pF for $\varepsilon_{\text{r}} = 4.6$, the 1.1 mm wafer being equivalent to a half-space within 0.01 %), and the beech values decrease monotonically with frequency, as required for a lossy dielectric, whereas the raw values rise between 20 and 100 kHz. Campaign rows: means of the conditioning stage (Table 1 and Section 3.2 of the main text) converted with the line of the respective meter; the drop of the glass reference at 100 kHz shows that LCR 2 operated with a smaller gain during the campaign (implied 1.09), whereas the calibrated dry beech value is 2.3 % higher at 100 than at 20 kHz, i.e., the gain of LCR 1 during the campaign was at least about 1.25 (lower bound for a passive dielectric). At 1 to 20 kHz, the campaign glass values lie 10 to 11 % below the model, i.e., the offsets during the campaign differed from those at calibration (LCR 2 read 0.68 to 0.73 pF lower). Dissipation factor at 100 kHz in this session: 0.03 (C0G) and 0.07 (glass), i.e., instrumental. A preliminary check of a 15 pF C0G capacitor in SLOW mode (1 October 2026) showed a different pattern (rise of 1.0 to 1.1 pF between 1 and 5 kHz, none between 20 and 100 kHz) and was not used; the speed setting thus changes the instrumental error. Data: `data/calibration_check.csv`.

	**1 kHz**	**5 kHz**	**20 kHz**	**100 kHz**
LCR 1 (beech): a (pF)	0.59	0.71	0.73	0.69
LCR 1: b	0.995	0.992	0.999	1.218
LCR 1: max. res. (pF)	0.04	0.03	0.01	0.07
LCR 2 (glass): a (pF)	0.48	0.62	0.64	0.70
LCR 2: b	1.003	0.997	1.005	1.232
LCR 2: max. res. (pF)	0.06	0.05	0.05	0.02
Glass reference, LCR 1 (pF)	9.28 / 8.73	9.32 / 8.68	9.35 / 8.63	11.30 / 8.71
Glass reference, LCR 2 (pF)	9.11 / 8.61	9.19 / 8.60	9.22 / 8.53	11.40 / 8.69
Beech sensor, LCR 1 (pF)	10.20 / 9.65	9.43 / 8.79	9.11 / 8.39	10.70 / 8.22
Beech sensor, LCR 2 (pF)	10.30 / 9.80	9.53 / 8.94	9.20 / 8.51	11.10 / 8.44
Campaign: glass, LCR 2 (pF)	8.40 / 7.90	8.51 / 7.91	8.49 / 7.81	9.32 / 7.00
Campaign: dry beech, LCR 1 (pF)	6.87 / 6.31	7.57 / 6.92	7.53 / 6.81	9.17 / 6.97

Table S4: Uncertainty budget of the 100 kHz capacitance of the beech sensor and noise analysis. Instrument specification of the HM8118 from the manufacturer's manual and data sheet (basic accuracy 0.05 % at 1 V, SLOW/MEDIUM mode; the FAST mode used here doubles the values); the region-dependent accuracy applies at the high impedances of this fixture. The specification does not cover the gain error found by the calibration at 100 kHz in the FAST mode (Table S3). The combined statement used in the main text is that the calibrated absolute values are uncertain by −0.1/+0.6 pF, dominated by a possible offset shift between campaign and calibration; sensitivities are affected by the gain uncertainty (a lower bound of the gain; −2.3 % or more with the offsets of the calibration, about −0.4 % with offsets shifted as observed for the glass meter) and, at 90 % RH, by a possible change of the offset (water film, about 0.2 pF).

Contribution	Basis	Magnitude
Meter accuracy, 1 kHz	$\|Z\|$ = 0.74–23 MΩ (beech; lower value in the wet stage A90, Table S7), 19 MΩ (glass); specification 0.2 % + $\|Z\|/1.5\,\mathrm{G\Omega}$ (manual, worked example for 10 pF at 1 kHz; SLOW/MEDIUM), doubled in the FAST mode at 1 V used here	0.5–3.5 %
Meter accuracy, 100 kHz	$\|Z\|$ = 0.09–0.17 MΩ; specification 0.5 % + $\|Z\|/100\,\mathrm{M\Omega}$ (accuracy chart of the data sheet, 10 to 100 kHz, $\|Z\| > 25\,\mathrm{k\Omega}$; no specification above 100 kHz for $\|Z\| > 25\,\mathrm{k\Omega}$), doubled in the FAST mode	1.2–1.3 % (0.12–0.21 pF)
Gain error at 100 kHz	C0G calibration (Table S3): $b = 1.218$ for the meter of the beech sensor (1.232 for the glass meter); removed by the calibration; consistent with the campaign data (dry beech: ratio of the 100 and 20 kHz readings after offset removal 1.25, a lower bound for a passive dielectric)	22 % (removed); residual −2.3 % or more with the offsets of the calibration, about −0.4 % with shifted offsets (campaign lower bounds 1.246 and 1.22; the gain of the glass meter changed from 1.09 to 1.232 between campaign and calibration)
Offset a (wires, pads)	C0G calibration: 0.69 pF at 100 kHz (0.48–0.73 pF at all frequencies and both meters); includes the lead capacitance of the wires (same wiring); removed by the calibration	0.69 pF (removed)
Offset during the campaign	not verifiable for the beech meter; the glass meter read 0.68 to 0.73 pF lower at 1 to 20 kHz during the campaign than at calibration; a like shift of the beech meter would raise its calibrated values by 0.71/1.218	+0.58 pF (one-sided; dry value 6.97 → 7.55 pF)
Difference between the meters	same sample on both meters after calibration: glass 0.03–0.12 pF, beech 0.12–0.22 pF	up to 3 %
Calibration fit and C0G tolerance	largest deviation from the fitted lines 0.07 pF; capacitor tolerance ±0.1 pF; readings at 100 kHz partly noted to 0.1 pF	≤0.1 pF
Glass-referenced corrections (superseded)	subtracting the rise of 0.92 pF of the glass reading between 1 and 100 kHz or dividing by the corresponding raw ratio of 1.109 (glass meter, conditioning stage; not to be confused with the offset-corrected campaign gain of 1.09); leave most of the gain error in the beech data: dry 8.26/8.27 pF, A90 16.45/15.66 pF	+1.3 pF (dry) against the calibration
Dissipation factor at 100 kHz	C0G capacitors and glass reference in the calibration session	0.03–0.07 (instrumental)
Three-dimensional end effects (model)	outer fingers (2D full comb vs. scaled unit cell); finger ends facing the opposite bus bar (estimate $24w/(23l)$); bus bars and pads not modeled	+1.9 %; ≈+2 %; unknown
Water film on the reference	transient rise of the glass reading in the 90 % RH stage (100 kHz)	up to 0.21 pF (2.3 %)
Reference step	single step of the glass reading at $t \approx 20$ h (1 kHz)	0.03 pF (0.4 %)
Tabulated permittivity (model)	assumed ±20 % in ε_r (Table S1)	±1.3/1.8/2.7 pF at 6.2/13.0/20.4 % MC
MC assignment (MC-referred values only)	tabulated EMC for Sitka spruce, midway between the branches; 1 percentage point at one or both ends of the 14.2-point span	7 or 14 % of S_MC
Noise, single reading	standard deviation in the last 16 h of conditioning ($n = 94$): beech 66 fF (100 kHz) and 39 fF (1 kHz), glass 3 fF and 4 fF; lag-1 autocorrelation 0.02, Ljung–Box $Q_6 = 2.6$ ($\chi^2_{6,0.95} = 12.6$); correlation with chamber RH $r = 0.13$ (RH s.d. 0.09 % RH) and temperature $r = -0.04$ (s.d. 0.03 °C); correlation with the glass reading $r = 0.66$, which points to a common external influence of small amplitude, whereas the 20-fold larger beech noise remains unexplained (separate meters, contact, lossy wood)	66 fF (raw), 55 fF (calibrated)
Noise, 1 h mean	standard deviation of the running mean of six consecutive readings	29 fF (raw), 24 fF (calibrated)

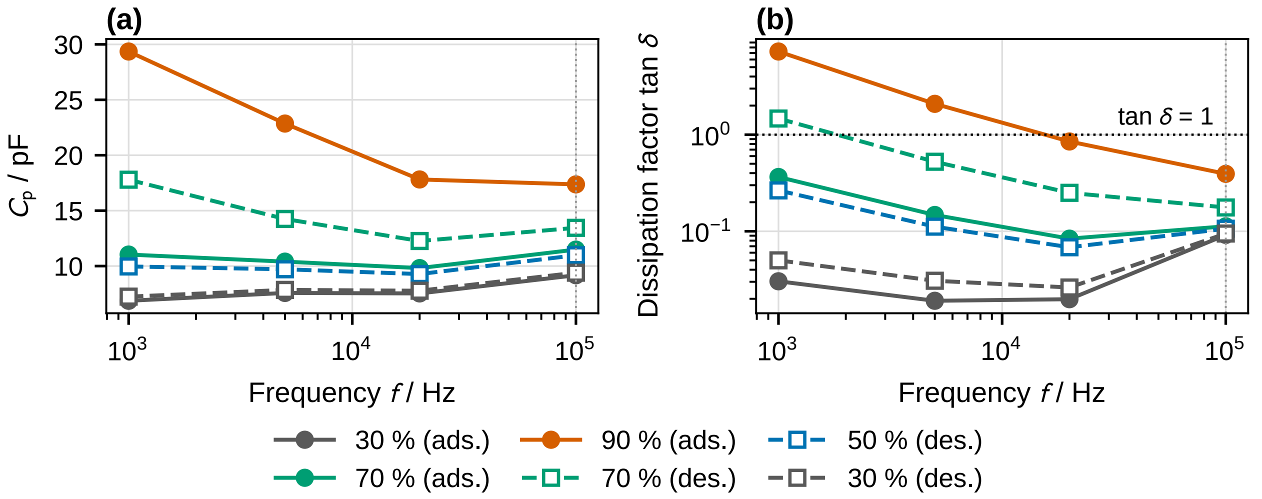

Figure S4: Frequency dependence of (**a**) the parallel capacitance and (**b**) the dissipation factor of the beech sensor for the stage end values of Table 1 of the main text (raw values; ads.: adsorption, des.: desorption branch). The raw 100 kHz values contain the gain error of 1.218 of the meter (Table S3). With $\tan\delta \gg 1$ at 1 kHz in the wet stages the displayed parallel capacitance is no longer a purely dielectric quantity; at 100 kHz, $\tan\delta \leq 0.39$ in all stages.

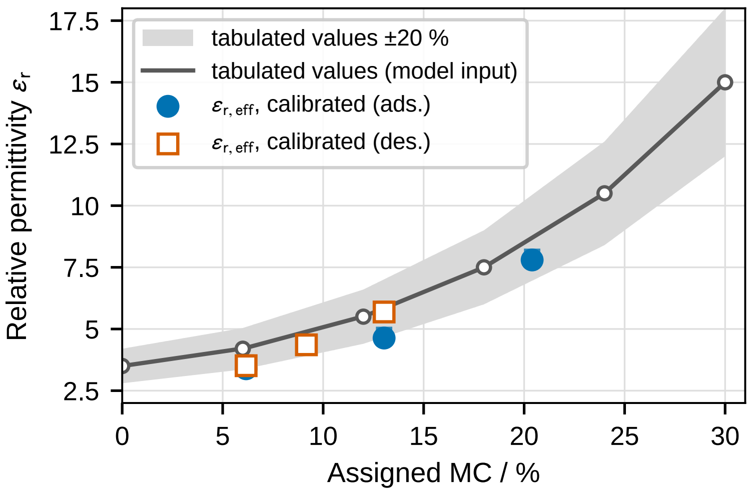

Figure S5: Effective permittivity of the wood back-calculated from the calibrated 100 kHz values via the linear model relation $C = 1.79\,\text{pF} + 1.53\,\text{pF} \cdot \varepsilon_r$ (error bars: $-0.1/{+0.6}$ pF uncertainty of the calibrated values, i.e., $-0.07/{+0.4}$ in ε_r), compared with the tabulated values used in the model and their ±20 % uncertainty band. This plot is a linear transformation of Figure 4a of the main text and not an independent test of the model.

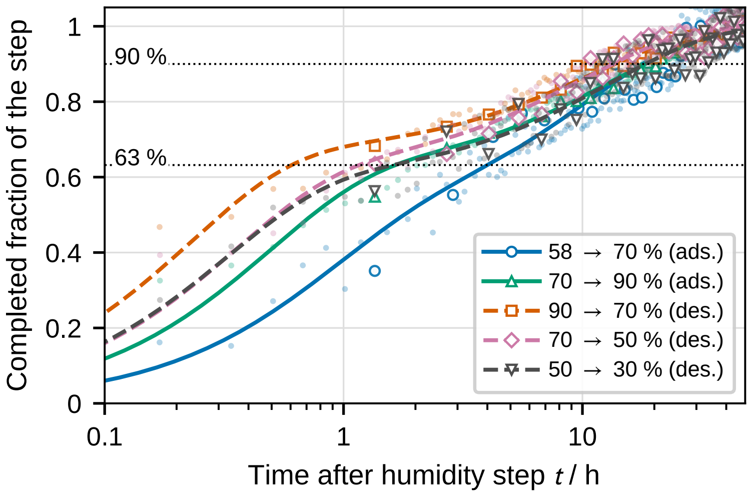

Figure S6: Step responses of Figure 4b of the main text (completed fraction of the step) on a logarithmic time axis (points; hollow markers every eighth reading) with the fitted two-phase curves (fixed C_0 and C_{end}); solid lines: adsorption, dashed lines: desorption; dotted lines mark 63% and 90% of the step.

Table S5: Step responses of the beech sensor at 100 kHz (raw values): step height ΔC, parameters of the two-phase fit $(C(t) - C_{end}) / (C_0 - C_{end}) = a\,e^{-t/\tau_s} + (1-a)\,e^{-t/\tau_l}$ with C_0 (mean of the 2 h before the step) and C_{end} (stage end value) fixed, with 95 % confidence intervals from the fit covariance; root-mean-square residual of the two-phase fit (RMS_2) and of a single-exponential fit with the same fixed end points (RMS_1); $\Delta AIC = AIC_1 - AIC_2$ (positive values favor the two-phase model); lag-1 autocorrelation of the residuals of the two-phase fit (r_1; the correlated residuals of the large steps stem from the first hour, Figure S2b); and model-free response times t_{63}, t_{90}, and t_{95} from 5-point median-filtered data. Because the residuals are autocorrelated, the ΔAIC values overstate the evidence for the two-phase model, and the confidence intervals are too narrow (by up to a factor of about 1.8 for $r_1 = 0.52$). The desorption values of τ_s are of the order of the chamber settling time and the sampling interval and are therefore not resolved ($\lesssim$ 0.5 h, Figure S2). The first adsorption step starts from the non-equilibrium state at the end of the ramp.

Step	ΔC (pF)	a	τ_s (h)	τ_l (h)	RMS_2 (fF)	RMS_1 (fF)	ΔAIC	r_1	t_{63} (h)	t_{90} (h)	t_{95} (h)
58 → 70 % RH (ads.)	1.38	0.47 ± 0.05	0.80 ± 0.24	10.7 ± 1.0	75	117	243	−0.03	3.5	15.4	20.4
70 → 90 % RH (ads.)	5.86	0.60 ± 0.01	0.47 ± 0.04	13.3 ± 0.5	113	529	877	0.52	2.4	18.7	29.5
90 → 70 % RH (des.)	−3.93	0.66 ± 0.01	0.22 ± 0.03	11.0 ± 0.6	97	331	691	0.37	1.4	10.7	19.8
70 → 50 % RH (des.)	−2.45	0.61 ± 0.02	0.34 ± 0.06	9.7 ± 0.7	78	201	533	0.13	1.4	10.8	14.4
50 → 30 % RH (des.)	−1.58	0.59 ± 0.03	0.32 ± 0.08	12.9 ± 1.0	73	160	447	0.14	2.2	13.2	17.0

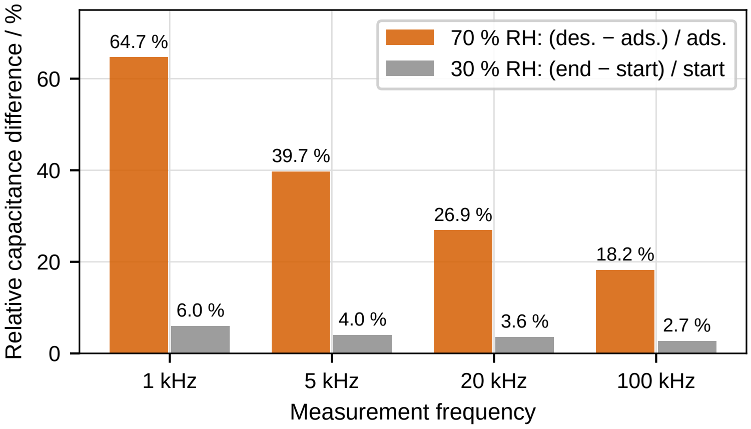

Figure S7: Relative difference between the desorption and adsorption end values at 70 % RH (des. − ads., referred to the adsorption value) and between the final and the initial 30 % RH state (end − start, referred to the initial value; both states approached by desorption) as a function of the measurement frequency. Both differences are computed from the calibrated values of the respective frequency (Table S3). The strong decrease with frequency shows that the low-frequency differences are dominated by the after-effect of the conduction-related polarization of the preceding wet stage; the 100 kHz values (18.2 %, rounded to 18 % in the main text, and 2.7 %) are the upper bounds of hysteresis and non-reversibility discussed in the main text.

Table S6: Capacitive wood-moisture sensors, printed sensors on wood, and printed capacitive humidity sensors compared with this work. Values as reported in the cited sources; n/s: not stated there. Sensitivities are given as reported; relative values only where the source reports them.

**Sensor / source**	**Substrate, sensing dielectric**	**Electrodes, fabrication**	**Readout**	**Range**	**Sensitivity**	**Response time**	***n***
This work	beech, solid wood (substrate = dielectric)	IDC, 24 fingers, $w = s = 300\,\mu m$, DIW silver paste, cured 150 °C	two LCR meters (HM8118), 100 kHz, calibrated with C0G capacitors	30–90 % RH (6.2–20.4 % MC assigned)	0.11 pF per pp RH (1.6 % of base capacitance; +97 % over 30–90 % RH); 0.47 pF per pp assigned MC (0.46–0.55 including gain and sorption-branch uncertainty, excluding the species-related EMC uncertainty)	t_{90} 11–19 h (two-phase; fast fraction not resolved for desorption)	1
Thakur et al. 2024 [S1]	pine wood (substrate = dielectric)	IDC drawn by hand with an 8B graphite pencil (designs without fingers and with 5 fingers); primarily touch sensors, humidity response reported	Keithley 4200-SCS, 25 mV; frequency n/s	30–90 % RH	relative change $\Delta C/C \approx$ 0.1 to 0.65 over 30–90 % RH (non-linear; reference state n/s)	n/s	n/s
Mulla et al. 2023 [S2]	spruce and pine wood	laser-graphitized lignin-based ink (bio-graphene), resistive	resistance	10–90 % RH at 25 °C	2.6 (spruce) and 0.74 (pine) MΩ per pp RH	n/s	n/s
Forsthuber et al. 2023 [S3]	Norway spruce blocks $150 \times 75 \times 20$ mm (substrate = dielectric)	interdigitated carbon-ink electrodes, inkjet-printed on a waterborne acrylic primer or screen-printed without primer, sintered 120 °C	LCR meter (HM8118), impedance and phase; best at 5 kHz, 1 V	50–98 % RH at 23 °C (EMC ≈ 9–25 %)	impedance regression on MC, $R^2 = 0.94$; no sensitivity per MC stated	n/s (climates held until equilibrium, about 3 weeks)	3 per variant
Chetpattananondh et al. 2017 [S4]	rubber wood (probe placed on the wood)	IDC on a circuit board, $50\,mm \times 73\,mm$, gap 2 mm	modified De Sauty bridge, 1 kHz	6–70 % MC	quadratic calibration C(MC); accuracy error 2 %, precision error 0.98 % (full scale)	n/s	35 + 120 wood samples, one probe
Korkua and Sakphrom 2020 [S5]	oil-palm wood (samples $8 \times 8 \times 1.5$ cm)	parallel plates 8×8 cm (3 cm apart) and coplanar plates 8×3 cm (2 cm apart)	square-wave excitation, 200 kHz	5–20 % MC	4–219 pF (plate) and 20–100 pF (coplanar) over the range; max. mean error 0.78 % (plate), 2.16 % (coplanar)	n/s	n/s
Laleicke and Kamke 2018 [S6]	maple veneers (calibration)	four-channel planar capacitance sensor with different spatial wavelengths	capacitance-to-digital converters	three RH levels (calibration)	MC gradient detection demonstrated	n/s	n/s
Li et al. 2018 [S7]	glued-laminated timber	pin-type or surface electrodes embedded inside or between the lamellas	electrical resistance or capacitance	10–70 % MC	n/s	n/s	n/s
Gaspar et al. 2017 [S8]	paper, 90 g/m^2 (substrate = sensing layer)	IDC, 40 fingers, 200 µm wide, gap 150 µm, inkjet silver	HP 4192A, 100 kHz	40–100 % RH	≈ 2 pF per pp RH	≈ 5 min (recovery < 4 min)	≥ 3
Rivadeneyra et al. 2014 [S9]	polyimide foil, 75 µm (foil = dielectric)	IDC, 63 fingers, 50 µm/50 µm, inkjet silver, 11.65 mm^2	Agilent 4294A, 100 kHz and 1 MHz, 0.5 V	10–98 % RH	4.5 fF per pp RH (100 kHz); thermal drift −0.4 fF per °C	356 s (ads.), 367 s (des.)	5

Table S7: Full impedance of the beech sensor at the stage end values of Table 1 of the main text (mean of the last 6 h before the next set-point change, 35 readings per frequency; raw values, i.e., without the calibration of Table S3; the 100 kHz values contain the gain error and an instrumental dissipation factor of about 0.03–0.07 (Table S3), so G_p and φ at 100 kHz are largely determined by the meter in the dry stages). C_p and R_p are the readings of the meter in the parallel equivalent circuit; $G_p = 1/R_p$, $\tan\delta = G_p/(\omega C_p)$, and the magnitude $|Z| = 1/\sqrt{G_p^2 + (\omega C_p)^2}$ and phase $\varphi = -\arctan(\omega C_p/G_p)$ of the impedance follow from them and are averaged reading by reading. In the wet stage A90, G_p changes by a factor of only 1.4 between 1 and 20 kHz, whereas C_p falls from 29.4 to 17.8 pF and the phase approaches 0° at 1 kHz: the low-frequency response is dominated by frequency-independent (dc-like) ionic conduction, which enters only G_p, and by the polarization it causes at interfaces and at the bare silver electrodes, which raises C_p; C_p there is an apparent quantity of the parallel representation.

**Phase**	**RH (%)**	***f* (kHz)**	**C_p (pF)**	**R_p (MΩ)**	**G_p (nS)**	**tan δ**	**$\|Z\|$ (kΩ)**	**φ (°)**
C (des.)	30	1	6.87	782	1.31	0.0304	23165	−88.3
		5	7.57	251	4.54	0.0191	4202	−88.9
		20	7.53	63.0	18.8	0.0198	1056	−88.9
		100	9.17	1.91	528	0.0917	173	−84.8
A70 (ads.)	70	1	11.04	39.5	25.3	0.365	13546	−70.0
		5	10.40	20.8	48.2	0.148	3028	−81.6
		20	9.82	9.71	103	0.0839	808	−85.2
		100	11.49	1.23	813	0.113	138	−83.6
A90 (ads.)	90	1	29.36	0.747	1340	7.26	740	−7.8
		5	22.86	0.668	1498	2.09	602	−25.6
		20	17.81	0.525	1904	0.851	340	−49.6
		100	17.37	0.233	4291	0.393	85.3	−68.5
D70 (des.)	70	1	17.79	6.09	164	1.47	5032	−34.2
		5	14.25	4.26	235	0.525	1978	−62.3
		20	12.26	2.60	385	0.250	630	−76.0
		100	13.45	0.671	1491	0.176	117	−80.0
D50 (des.)	50	1	9.96	60.3	16.6	0.265	15440	−75.2
		5	9.71	29.4	34.1	0.112	3258	−83.6
		20	9.28	12.6	79.7	0.0683	856	−86.1
		100	10.98	1.37	733	0.106	144	−83.9
D30 (des.)	30	1	7.24	443	2.28	0.0500	21952	−87.1
		5	7.85	139	7.58	0.0308	4054	−88.2
		20	7.77	42.8	25.6	0.0262	1023	−88.5
		100	9.40	1.80	560	0.0948	169	−84.6

References (Supplementary Materials)

[S1] Thakur, A.S.; Srivatava, V.; Park, H.K.B.; Kebaili, I.; Boukhris, I.; Joo, Y.H.; Sung, T.H.; Kumar, A.; Vaish, R. Calligraphic interdigitated capacitive sensors for green electronics. *Sci. Rep.* **2024**, *14*, 15685. `https://doi.org/10.1038/s41598-024-64461-2`

[S2] Mulla, M.Y.; Isacsson, P.; Dobryden, I.; Beni, V.; Östmark, E.; Håkansson, K.; Edberg, J. Bio-graphene sensors for monitoring moisture levels in wood and ambient environment. *Glob. Chall.* **2023**, *7*, 2200235. `https://doi.org/10.1002/gch2.202200235`

[S3] Forsthuber, B.; Truskaller, M.; Spitaler, I.; Kulha, P.; Scherf, M.; Partl, G.; Rössler, A.; Engelbrecht, G.; Grüll, G. Printed sensors for monitoring wood moisture content inside timber building elements. In Proceedings of the World Conference on Timber Engineering (WCTE 2023), Oslo, Norway, 2023; pp. 3874–3879. `https://doi.org/10.52202/069179-0504`

[S4] Chetpattananondh, P.; Thongpull, K.; Chetpattananondh, K. Interdigital capacitance sensing of moisture content in rubber wood. *Comput. Electron. Agric.* **2017**, *142*, 545–551. `https://doi.org/10.1016/j.compag.2017.11.016`

[S5] Korkua, S.K.; Sakphrom, S. Low-cost capacitive sensor for detecting palm-wood moisture content in real-time. *Heliyon* **2020**, *6*, e04555. `https://doi.org/10.1016/j.heliyon.2020.e04555`

[S6] Laleicke, P.F.; Kamke, F.A. A capacitive multi-wavelength sensor for moisture content gradient sensing in wood. *Wood Sci. Technol.* **2018**, *52*, 717–732. `https://doi.org/10.1007/s00226-018-0988-z`

[S7] Li, H.; Perrin, M.; Eyma, F.; Jacob, X.; Gibiat, V. Moisture content monitoring in glulam structures by embedded sensors via electrical methods. *Wood Sci. Technol.* **2018**, *52*, 733–752. `https://doi.org/10.1007/s00226-018-0989-y`

[S8] Gaspar, C.; Olkkonen, J.; Passoja, S.; Smolander, M. Paper as active layer in inkjet-printed capacitive humidity sensors. *Sensors* **2017**, *17*, 1464. `https://doi.org/10.3390/s17071464`

[S9] Rivadeneyra, A.; Fernández-Salmerón, J.; Agudo, M.; López-Villanueva, J.A.; Capitan-Vallvey, L.F.; Palma, A.J. Design and characterization of a low thermal drift capacitive humidity sensor by inkjet-printing. *Sens. Actuators B Chem.* **2014**, *195*, 123–131. `https://doi.org/10.1016/j.snb.2013.12.117`

[S10] Mamishev, A.V.; Sundara-Rajan, K.; Yang, F.; Du, Y.; Zahn, M. Interdigital sensors and transducers. *Proc. IEEE* **2004**, *92*, 808–845. https://doi.org/10.1109/JPROC.2004.826603

[S11] Glass, S.V.; Zelinka, S.L. Moisture relations and physical properties of wood. In *Wood Handbook—Wood as an Engineering Material*; General Technical Report FPL-GTR-282; U.S. Department of Agriculture, Forest Service, Forest Products Laboratory: Madison, WI, USA, 2021; Chapter 4.
